# University teachers’ scientific research innovation incentive based on the three-party evolutionary game of the state, the colleges, and scientific researchers

**DOI:** 10.3389/fpsyg.2022.973333

**Published:** 2023-01-12

**Authors:** Yan Zheng

**Affiliations:** School of Business Administration, Nanchang Institute of Technology, Nanchang, China

**Keywords:** dynamic stability, evolutionary game theory, numerical simulation, scientific innovation, scientific research

## Abstract

Scientific research in colleges and universities is of great significance to national innovation. Based on the evolutionary game theory, this paper constructs a theoretical model of the state, universities, and researchers. This paper also conducts numerical simulation on the model. The results reveal that when the scientific researchers’ success rate reaches a certain threshold, more and more scientific researchers will choose to invest in scientific research. Then, universities and the state will hold a long-term incentive attitude toward scientific research and scientific innovation. The study further found that the greater the success rate of researchers, the faster universities and the state will actively encourage scientific research.

## 1. Introduction

Innovation promotes the development of social productive forces. Innovation promotes changes in production relations and social systems. Innovation promotes the development of human thinking and culture. Talent is the core element of innovation. To give full play to the core role of talent in innovation-driven, it is necessary to firmly establish the strategic position of talent to lead development, gather talents in all directions, and strive to consolidate the talent foundation for innovation and development. That innovation-driven development has become an important strategy of national development and placed in the primary position.

In the new era of innovation-driven development, as one of the important subjects of knowledge innovation, colleges and universities play an irreplaceable role in promoting national development, social and economic progress, and industry promotion. From the perspective of social development and economic progress, scientific research innovation in colleges and universities plays a great role in promoting the collaborative innovation of colleges and universities, scientific research institutions and industries, as well as the innovation of academic start-up enterprises, and the reform and breakthrough of the new generation of information technology industry (Zuchao and Chunxiao, 2012; Haiyan and Huawei, 2020). From the perspective of talent training and social progress, scientific research innovation in Colleges and universities can play a positive driving role in the training of innovative talents (Chuanzhong and Zhipeng, 2019). However, at present, the efficiency of scientific research and innovation in Colleges and universities in China is generally low, showing a slight growth trend under the background of “double first-class” (Eryong, 2012; Ning and Luyu, 2019; Hui and Olympic, 2020). Further research shows that the investment of scientific research human capital in colleges and universities in China is generally “scarce,” while the material capital represented by funds is in the “redundant” stage (Hui and Olympic, 2020). Ning and Luyu (2019) found that there is room for improvement in the scientific research efficiency of 40 “first-class universities,” and the utilization rate of scientific research resources, scientific research quality, and transformation of scientific research achievements need to be improved.

## 2. Literature review

### 2.1. Theoretical underpinnings

Evolutionary game theory can be traced back to Scottish moral philosophy led by Ferguson, Hume, Mondewell, and Smith. In the German historical school and Marxist economics, evolutionary thinking is widely used to analyze the changes of social and economic structures. With the further promotion of Darwinism, the evolution theory has developed more rapidly (Kainan, 2009).

According to evolutionary game theory, under the rule of survival of the fittest in nature, individual organisms will imitate the behavior of species whose income is above the average level in the group, adopt the survival rule that most suitable for nature, change their original living habits and behaviors, and promote the group behavior of species to reach equilibrium.

Biologists try to use game theory to build various models of biological competition and evolution (Lewontin, 1961). Combined with the principle of biological evolution, biologists introduced the payment function and evolutionary stability strategy into the traditional game theory (Maynard Smith and Price, 1973), and thus constructed a replication dynamic model. (Taylor and Jonker, 1978; Maynard Smith, 1982).

Evolutionary game theory combines the “equilibrium view” in economics with the “adaptability” in biology. Under the conditions of incomplete rationality, asymmetric information, and deviation from others’ behavior expectations, evolutionary game describes the process and results of people’s continuous response to the impact of the outside world through imitation, learning, trial and error (Qingwen et al., 2003). Evolutionary game theory believes that when a player encounters a complex problem, his perception will generally trigger his behavior, and he will imitate the behavior of a successful person. This behavior is very similar to the behavior of other creatures, as well as the competition and cooperation behavior of human beings. Game is a dilemma. Static game cannot fully explain the occurrence of the dilemma. On the contrary, dynamic factor (replication) refers to the influence of strategic allocation on the dilemma by influencing game dynamics (Tanimoto and Sagara, 2007). Using the analytical framework of evolutionary game theory, Wang et al. (2015) and Ito and Tanimoto (2018) effectively proved that natural selection would be beneficial to the group cooperation of selfish individuals.

The hypothesis of human in evolutionary game theory is “limited and rational people.” Limited rationality is expressed as a dynamic evolutionary behavior choice determined by individuals in their understanding and learning of the game environment. This kind of behavior choice is to make behavior judgments and behavior decisions for the players from the positive side, so that the players can realize the target income of individuals in the process of continuous learning and imitation, and finally achieve the dynamic equilibrium of the game group (Xianjia et al., 2011).

As time goes by, evolutionary game theory has been used to analyze many social and economic problems (Arefin et al., 2020). Evolutionary game theory regards human behavior choice as a strategy choice. Due to the incompleteness of information, evolutionary game theory analyzes the evolution trend of the individual behavior of limited rationality in groups affected by groups. Based on the limited rationality of human beings, the players will constantly adjust and change their strategies and behavior choices according to the constant changes and situations of society, so as to make the behaviors of each player reach Nash equilibrium. In addition, this method is also used to widely analyze other social problems, such as the development of epidemic diseases (Tanimoto, 2021).

Evolutionary game model must meet four main conditions to ensure that all survivors’ behavior is profit maximization. The four conditions are diversity, continuity of behavior, profit driven growth and limited path dependence (Nelson and Winter, 2002). Diversity refers to biological diversity, that is, multiple players; Behavior continuity refers to the game players’ behavior strategies that are constantly changing according to the changing environment, that is, the replication dynamic equation constructed by differential equation; The growth caused by profits refers to the gains pursued by the players; Effective path dependence means that under the premise of limited rationality, the players consider the limited benefits, but not all benefits.

When using evolutionary game theory to analyze social phenomena, the government plays a very important role in the game process. For example, Md et al. (2022) analyzed the behavior strategy choice of photovoltaic power plants and coal-fired power plants with government subsidies in great detail. Yan and Xiaoming (2019) also found that the government plays an important role in the game. Therefore, this study uses the government as the game player.

### 2.2. Scientific research innovation and innovation ability

#### 2.2.1. Scientific research innovation

Innovation refers to invention, innovation, imitation and transcendence. Innovation is defined as “new academic ideas, new scientific discoveries, new technological inventions, and new industrial directions” (Ziyang et al., 2022). Innovation characterized by technology and knowledge requires researchers to invest a lot of time, energy and money. Once the innovative achievements are formed, they will greatly promote the society, the country and the economy. The innovative achievements belong to public goods and have positive external benefits. However, the innovative achievements are easy to be imitated and surpassed by competitors, which requires appropriate protection and support from relevant departments (governments, universities) to promote the sustainable development of innovation. The research shows that the average time from the introduction of new products by enterprises to the provision of the same or similar products by competitors has decreased from more than 30 years in the early 20th century to more than 3 years at the end of the 20th century (Agarwal and Gort, 2001). Therefore, the spillover effect of enterprise R&D has damaged the enthusiasm of competitive enterprises to carry out R&D activities to a certain extent, leading to the transformation of R&D competition into a “waiting game,” that is, enterprises do not think about independent R&D, but only want to “hitchhike” and enjoy their success (Yang, 2007). The external spillover effect of enterprise innovation is manifested in all stages of R&D, and the innovation efficiency of each stage has obvious spatial correlation (Yongze and Dayong, 2013). This kind of innovation spillover also exists in universities and research institutions. Most studies show that the innovation output of universities and research institutions has an obvious spillover effect on enterprise innovation (Jaffe, 1989; Fritscha and Frankeb, 2004). The spillover of R&D innovation can be adjusted through national and government policies. For enterprises with R&D behavior, the government’s special subsidy for scientific research has a significant positive incentive effect on the R&D of enterprises in the next year (Jiuqin and Dandan, 2011).

To sum up, the scientific research innovation of enterprises and university researchers has positive externalities. This positive externality can optimize its social benefits through relevant policy adjustments. Adjustment means include certain R&D subsidies or special preferential policies given to R&D personnel by the government, universities and other departments. The influence of the state on university researchers is macro, while that of universities is micro. Therefore, this study chooses two subjects, the state and universities, to analyze the impact of national and university policies on the research behavior of university researchers.

#### 2.2.2. Innovation ability

On the one hand, innovation ability is related to individual factors. Innovation ability is a process in which people create new ideas or new methods to solve problems in the process of learning, production and research, and make efforts to realize the ideas and methods (Xianwei and Yonghong, 2017). The process of innovation is a subjective dynamic process of people, and the subjective will of people plays a decisive role in the process of innovation. People’s psychological capital (hope, optimism, tenacity) affects individual behavior, and has a positive correlation with employees’ job performance and organizational commitment (Lifeng, 2007). That is, a positive mental state will improve work performance. Wang et al. (2022) also found that there is a significant positive correlation between the dimensions of psychological capital and entrepreneurial performance. There is a positive correlation between college teachers’ personal scientific research efficacy and their scientific research performance (Hemmings and Kay, 2016), and the research of Pasupathy and Siwatu (2014) has reached a similar conclusion.

Tom et al. (2006) believes that the incentive to university scientific researchers can improve scientific research performance, but when scholars realize this demand, the incentive effect of the original incentive measures on scientific research innovation will be weakened. Guang and Huajun (2019) found that the results of scientific research performance are mainly affected by scientific research attitude and scientific research behavior; salary, assessment, achievement, and other incentive methods will not affect the attitude and behavior of scientific research, while promotion, innovation, social networking, and other incentive methods will not only affect the scientific research process but also affect the scientific research results through the process.

Scientific research performance is also related to external pressure. Performance pressure has a positive and negative boundary effect on teachers’ research behavior. When employees regard performance pressure as a challenge, factors such as job remodeling, job involvement, task proficiency, performance improvement and creativity will have a positive effect on performance pressure (Suosuo et al., 2022).

The time input will also affect the scientific research performance of university teachers. There is an inverted U-shaped relationship between work time (work engagement) and work performance (Qiang et al., 2014). This inverted U-shaped relationship is more significant in China, in knowledge intensive industries, in low age groups, and in male groups (Haojie et al., 2022).

The creativity of college teachers will also affect their scientific research ability. Compared with those with low creativity, those with high creativity have higher cognitive inhibition ability, which can effectively inhibit the reaction tendency unrelated to advantages (Xuejun and Haijuan, 2018).

On the other hand, innovation ability is related to external factors. Organizational management system and organizational support affect the sense of scientific research efficacy, and then have a significant positive impact on scientific research innovation (Lichao and Haohao, 2022). Rui et al. (2022) believed that innovation guarantee, innovation environment and other factors had an impact on the innovation ability of university researchers. Qing et al. (2022) found that the assessment system, incentive policy, organizational support, and scientific research atmosphere in the organization are important external factors that affect the research passion of university researchers. The salary incentive, growth incentive and work incentive in the organization can play a positive role in the research personnel of public welfare research institutes (Xinwei, 2010). When formulating the salary incentive system, we should pay attention to the combination of short-term and long-term benefits of scientific researchers, and adjust the reasonable proportion of salary performance intensity to produce more breakthrough results (Lirong and Haitao, 2012).

Nir and Ailberstein (2006) also believes that incentive methods such as professional title promotion, evaluation, and employment make scientific research staff blindly pursue the number of papers and patents and ignore scientific research ethics and research ethics. Especially under the realistic condition of fierce promotion of professional titles, incentive measures related to professional titles hurt the innovation performance of scientific researchers (Yoon, 2016).

Inclusive leadership has a significant positive impact on the innovation performance of researchers, and the sense of responsibility significantly regulates the relationship between inclusive leadership and innovation performance of researchers (Xiaomei et al., 2016; Gupta et al., 2022). Qingjin et al. (2010) also found that a loose, harmonious, free, open and inclusive scientific research environment is conducive to improving the output of innovative achievements. Teoh et al. (2022) point out that idealized influence and inspirational motivation, have a significant positive influence on employee performance.

In terms of innovation performance of scientific research teams in colleges and universities, Haiyan (2015) found that the current management of scientific research teams in Colleges and universities ignores the characteristics of scientific research teams. It is suggested to improve the innovation ability of scientific research teams in colleges and universities from the aspects of innovative topics, talent training, team construction, and environmental construction. Xi and Huan (2019) found that the current implementation of the long-term employment system does not have a significant incentive effect on the academic output of young teachers’ scientific research team, while the academic output quality of young teachers under the renewal system has been significantly improved, but the promotion effect of strict renewal system assessment on the publication of engineering papers is significantly weaker than that of non-engineering majors (Yoon, 2016).

In terms of industry-university cooperation and scientific research performance, Qiang et al. (2019) found that the breadth of industry-university cooperation channels had a significant inverted U-shaped development trend on the scientific research performance of colleges and universities. The depth of industry-university cooperation channels can adjust the relationship between the breadth of industry-university cooperation channels and the scientific research performance of colleges and universities. With the increase of the depth of industry-university cooperation, the relationship between industry-university cooperation channels and scientific research performance gradually changes from an inverted U-shaped relationship to a positive correlation (Nir and Ailberstein, 2006).

### 2.3. Evolutionary game and teachers’ scientific research

Evolutionary game theory can be used to analyze teachers’ scientific research. (1) The core of evolutionary game theory is man’s “bounded rationality.” The core of evolutionary game theory is that as time goes on, individual behavior strategy changes and their final behavior choices are individual behavior strategy choices based on group income perception and comparison, and are behavior strategy choices of “limited and rational people.” (2) The essence of university teachers’ behavior strategy choice is human’s “limited rationality.” College teachers’ time, energy and information are limited. The core of the theory of “limited rationality” is that people’s time, energy and information are limited.

However, there are few literatures on the research innovation of university teachers using evolutionary game theory. With two players, Yaokun et al. (2022) and Xuwang et al. (2019) analyzed the theme of “evolutionary game” and “scientific research collaboration on academic social platforms.”

As pointed out above, innovation has positive externalities and needs the support of the state and government. Therefore, this paper takes the country, universities and researchers as the game players. Assuming that university teachers have scientific research innovation behavior, not all scientific research innovation behaviors will be successful, so this study increases the probability of scientific research success. Other assumptions of this study are different from the existing literature, and the conclusions of this study are new.

To sum up, from the perspective of “limited and rational people,” this paper analyzes the dynamic influence, evolutionary process, final behavior strategy selection and dynamic stability of university researchers by using the theory of tripartite evolutionary game. The theoretical contribution of this paper is to study the incentive mechanism of scientific research innovation in universities from different research perspectives and different research methods. The practical contribution lies in providing policy reference for scientific research management in colleges and universities.

## 3. Materials and methods

### 3.1. Parameters

*H1*: suppose there are three game players, namely, the state, university managers, and university researchers. As a natural person, the state has two strategic choices (high investment or low investment in scientific research innovation). University managers also have two strategic choices (positive incentive or negative incentive to university researchers). Researchers also have two strategic choices (positive responses or negative responses).

*H2*: when the state has a high investment in scientific research innovation in colleges and universities, and scientific researchers actively participate, the state will get huge benefits $V_{1}$ from scientific research and innovation. These benefits include the overall development of the state and the improvement of people's welfare. However, the state's high investment in scientific research needs a certain pay $C_{1}$ . If the state has high investment but the scientific researchers participate passively, the state will not only lose its high investment $\;C_{1}$ , but also suffer losses $M_{1}$ due to a lack of sufficient scientific research and innovation in the future. If the state does not make a high investment in scientific research but holds a negative attitude, the state will suffer huge losses $\;L_{1}$ due to the lack of scientific research and innovation in the future. (The difference between $L_{1}$ and $M_{1}$ is that some scientific research innovations $\;M_{1}$ may occur under the national active policy.)

*H3*: when colleges and universities actively encourage, and researchers respond positively，colleges and universities will obtain great benefits $\;V_{2}$ from scientific research innovation. These benefits include the improvement of the overall strength of colleges and universities, the enhancement of the comprehensive competitiveness of colleges and universities, the improvement of the national influence of colleges and universities, etc. At this time, the incentive of colleges and universities to scientific researchers needs to pay a certain cost $\;C_{2}$ . These costs include salary incentives and promotion incentives. If colleges and universities actively encourage, but researchers respond negatively, colleges and universities will suffer future development losses $\;M_{2}$ . If colleges and universities adopt negative policies toward scientific research, they will also suffer other losses $\;L_{2}$ . Other losses include the low comprehensive ranking caused by weak scientific research ability, the decline of the influence of colleges and universities, and the frustration of college enrollment and employment. (The difference between $\;L_{2}$ and $\;M_{2}$ is the same as $\;L_{1}\;$ and $\;M_{1}$ .)

*H4*: if researchers actively respond to the incentive policies, they will obtain certain benefits $\;C_{2}$ . These benefits are matched with the incentive cost of colleges and universities. In addition, researchers will also gain a good national reputation for their strong scientific research ability $\;R_{3}$ . But researchers also need to pay more time, energy, and mental labor cost $\;C_{3}$ . If researchers respond negatively to the university's scientific research incentives, or researchers fail to complete the university's scientific research tasks, they will suffer certain losses $\;L_{3}$ . These losses may be either salary deduction or demotion.

*H5*: the probability of high national investment in university scientific research innovation is x , the probability of positive incentive of university managers to university teachers' scientific research is y , the probability of positive response of scientific researchers to university scientific research incentive is z , and the probability of scientific researchers completing scientific research tasks under university scientific research incentive is θ . Table 1 is for all variable symbols and definitions, and assumptions.

**Table 1:** Definition and assumptions of the variables and relative conditions.

Symbol	Definition	Assumptions
x	High probability of national investment in scientific research and innovation in colleges and universities.	1>x>0
y	The probability of university administrators actively encouraging university researchers.	1>y>0
z	Probability of researchers’ positive response to scientific research incentive.	1>z>0
θ	Probability of scientific research personnel’s success in scientific research innovation.	1>θ>0
$V_{1}$	The value of scientific research and innovation to the state.	$V_{1}>0$
$C_{1}$	National investment cost of scientific research and innovation in colleges and universities.	$C_{1}>0$
$L_{1}$	Losses caused by the negative attitude of the state toward scientific research and innovation.	$L_{1}>0,$ $L_{1}>C_{1}$
$M_{1}$	Losses suffered by the state due to lack of scientific research and innovation achievements.	$M_{2}>0$
$V_{2}$	Benefits of scientific research innovation to colleges and universities.	$R_{3}>0$
$C_{2}$	The cost of scientific research incentive for teachers in colleges and universities = the comprehensive reward for teachers to achieve expected scientific research achievements, For example, for direct material rewards and hidden material rewards such as professional title promotion, most colleges and universities will give corresponding rewards according to the completion of teachers’ scientific research, rather than one-time rewards.	$C_{2}>0$
$L_{2}$	All kinds of losses caused by negative scientific research incentives for teachers in colleges and universities.	$L_{2}>0,L_{2}>C_{2}$
$M_{2}$	Losses suffered by colleges and universities due to lack of scientific research achievements.	$M_{2}>0$
$R_{3}$	After obtaining substantial innovation achievements, scientific researchers will receive invisible material rewards in terms of national reputation and so on.	$R_{3}>0$
$C_{3}$	All the efforts that researchers must make to achieve innovative results, even if they do, they may not be able to achieve the expected results.	$C_{3}>0$
$L_{3}$	In the case of high investment in scientific research by the state of colleges and universities, teachers respond negatively to all kinds of losses in scientific research. When the state and universities do not pay attention to scientific research and innovation, college teachers will not suffer losses if they do not carry out scientific research, but they will not benefit.	$L_{3}>0$

When the state actively encourages scientific research, if scientific researchers also actively carry out scientific research and innovation, whether colleges and universities actively encourage scientific research or not, the state will receive corresponding returns, and its comprehensive income is $\theta V_{1}-C_{1}$ . When the state actively encourages scientific research, if researchers treat scientific research negatively, regardless of whether colleges and universities actively encourage scientific research, the comprehensive income of the state is $-C_{1}-M_{1}$ .

When the state treats scientific research negatively, if researchers actively carry out scientific research and innovation, whether colleges and universities actively encourage scientific research or not, the state will also obtain certain benefits from teachers’ active scientific research $\theta V_{1}-L_{1}$ . When the state treats scientific research negatively, if researchers also treat scientific research negatively, whether colleges and universities actively encourage scientific research or not, the comprehensive income of the state is $-L_{1}-M_{1}$ .

When colleges and universities actively encourage scientific research, if researchers also actively carry out scientific research and innovation, whether the state actively encourages scientific research or not, colleges and universities will pay corresponding incentive costs according to the completion of scientific research projects, and therefore obtain corresponding returns. At this time, the comprehensive income of colleges and Universities is $\theta V_{2}-\theta C_{2}$ . When colleges and universities actively encourage scientific research, if researchers treat scientific research negatively, no matter whether the state actively encourages scientific research or not, colleges and universities do not obtain the benefits of scientific research innovation. At the same time, because researchers do not achieve scientific research results, colleges and universities do not pay the corresponding scientific research reward cost. However, at this time, colleges and universities suffer future development losses due to lack of scientific research and innovation, and their comprehensive income is $-M_{2}$ .

When colleges and universities treat scientific research negatively, if scientific researchers actively carry out scientific research and innovation, at this time, whether the state actively encourages scientific research or not, colleges and universities will obtain corresponding benefits because of the achievements made by scientific researchers. At the same time, because colleges and universities treat scientific research negatively, it will have a long-term negative impact and cause losses to the future development of colleges and universities. At this time, the comprehensive income of colleges and universities is $\theta V_{2}-L_{2}$ . When colleges and universities treat scientific research negatively, if researchers also treat scientific research negatively, no matter whether the state actively encourages scientific research or not, colleges and universities will bear the losses caused by the lack of scientific research achievements $M_{2}$ . At the same time, it will also bear the long-term negative losses caused by the negative treatment of scientific research $L_{2}$ . At this time, the comprehensive income of colleges and universities is $-L_{2}-M_{2}$ .

When teachers actively participate in scientific research and universities and the state encourages scientific research innovation, teachers will receive rewards from the state and universities according to the completion of scientific research achievements, but researchers need to pay a certain cost of time and energy for scientific research. At this time, the comprehensive income of researchers is $\theta \left(C_{2}+R_{3}\right)-C_{3}$ . When teachers actively participate in scientific research, the state also encourages scientific research, but colleges and universities treat scientific research negatively, at this time, scientific researchers can only get some rewards from the state according to the completion of scientific research achievements. In the research process, researchers also need to pay a certain cost of time and energy. At this time, the comprehensive income of researchers is $\theta R_{3}-C_{3}$ . When teachers actively participate in scientific research, colleges and universities also encourage scientific research, but the state treats scientific research negatively, at this time, researchers can only get some rewards from colleges and universities according to the completion of scientific research achievements. In the research process, researchers also need to pay a certain cost of time and energy. At this time, the comprehensive income of researchers is $C_{2}-C_{3}$ . When teachers actively participate in scientific research and universities and the state treat scientific research negatively, researchers cannot get any benefits at this time. Only pay purely in the process of research. At this time, the comprehensive income of researchers is $-C_{3}$ .

When teachers treat scientific research negatively and actively encourage scientific research innovation in colleges and universities or the state, teachers will hurt themselves because of their negative treatment of scientific research and need to bear certain losses. At this time, the comprehensive income of scientific researchers is $-L_{3}$ . When teachers treat scientific research negatively, if the state and universities also treat scientific research negatively, at this time, researchers have no income and loss, and their comprehensive income is 0. Table 2 is for the income matrix of the state, universities, and researchers.

**Table 2:** Income matrix of countries, universities, and researchers.

		Scientific researchers z	Scientific researchers ( 1−z )
State x	Colleges and universities y	$\theta V_{1}-C_{1}$ , $\theta V_{2}-\theta C_{2}$ , $\theta \left(C_{2}+R_{3}\right)-C_{3}$	$-C_{1}-M_{1}$ , $-M_{2}$ , $-L_{3}$
	Colleges and universities ( 1−y )	$\theta V_{1}-C_{1}$ , $\theta V_{2}-L_{2}$ , $\theta R_{3}-C_{3}$	$-C_{1}-M_{1}$ , $-L_{2}-M_{2}$ , $-L_{3}$
State ( 1−x )	Colleges and universities y	$\theta V_{1}-L_{1}$ , $\theta V_{2}-\theta C_{2}$ , $\;\theta C_{2}-C_{3}$	$-L_{1}-M_{1}$ , $-M_{2}$ , $-L_{3}$
	Colleges and universities ( 1−y )	$\theta V_{1}-L_{1}$ , $\theta V_{2}-L_{2}$ , $-C_{3}$	$-L_{1}-M_{1}$ , $-L_{2}-M_{2}$ , 0

### 3.2. Model construction, solution, and analysis

Assuming that the state actively encourages scientific research innovation and makes a large amount of investment, the expected return is $\;U_{1C}$ , the expected return of choosing negative encouragement and not paying attention to scientific research investment is $\;U_{1n}$ , the average expected return is $\bar{U_{1}}$ , Get:


(1) $$\{\begin{array}{c} \begin{array}{l} U_{1C}=\left(\theta V_{1}-C_{1}\right)yz+\left(-C_{1}-M_{1}\right)y\left(1-z\right)+\\ \left(\theta V_{1}-C_{1}\right)\left(1-y\right)z+\left(-C_{1}-M_{1}\right)\left(1-y\right)\left(1-z\right) \end{array}\\ \begin{array}{l} U_{1n}=\left(\theta V_{1}-L_{1}\right)yz+\left(-L_{1}-M_{1}\right)y\left(1-z\right)+\\ \left(\theta V_{1}-L_{1}\right)\left(1-y\right)z+\left(-L_{1}-M_{1}\right)\left(1-y\right)\left(1-z\right)\; \end{array}\\ \bar{U_{1}}=xU_{1C}+\left(1-x\right)U_{1n}\; \end{array}\;$$

According to Equation (1), the state actively encourages scientific research innovation and makes a large amount of investment to copy the dynamic equation:


(2) $$F\left(x\right)=\frac{d_{x}}{d_{t}}=x\left(U_{1c}-\bar{U_{1}}\right)=x\left(1-x\right)\left(L_{1}-C_{1}\right)\;$$

Suppose that the expected income of actively encouraging scientific research in Colleges and universities is $\;U_{2C}$ , the expected return of negatively encouraging scientific research is $\;U_{2n}$ , the average expected return is $\bar{U_{2}}$ ， Get:


(3) $$\{\begin{array}{c} \begin{array}{l} U_{2C}=\left(\theta V_{2}-\theta C_{2}\right)xz+\left(-M_{2}\right)x\left(1-z\right)+\\ \left(\theta V_{2}-\theta C_{2}\right)\left(1-x\right)z+\left(-M_{2}\right)\left(1-x\right)\left(1-z\right)\; \end{array}\\ \begin{array}{l} U_{2n}=\left(\theta V_{2}-L_{2}\right)xz+\left(-L_{2}-M_{2}\right)x\left(1-z\right)+\\ \left(\theta V_{2}-L_{2}\right)\left(1-x\right)z+\left(-L_{2}-M_{2}\right)\left(1-x\right)\left(1-z\right)\; \end{array}\\ \bar{U_{2}}=yU_{2C}+\left(1-y\right)U_{2n}\; \end{array}\;$$

According to Equation (3), the replication dynamic equation of actively encouraging scientific research in Colleges and universities is obtained:


(4) $$F\left(y\right)=\frac{d_{y}}{d_{t}}=y\left(U_{2c}-\bar{U_{2}}\right)=y\left(1-y\right)\left(L_{2}-\theta C_{2}z\right)$$

Suppose that the expected income of University researchers actively carrying out scientific research is $\;U_{3C}$ , the expected return of passive scientific research is $\;U_{3n}$ , the average expected return is $\bar{U_{3}}$ ， Get:


(5) $$\{\begin{array}{c} \begin{array}{l} U_{3C}=\left[\theta \left(C_{2}+R_{3}\right)-C_{3}\right]xy+\left(\theta R_{3}-C_{3}\right)x\left(1-y\right)+\\ \left(\theta C_{2}-C_{3}\right)\left(1-x\right)y+\left(-C_{3}\right)\left(1-x\right)\left(1-y\right) \end{array}\\ U_{3n}=\left(-L_{3}\right)xy+\left(-L_{3}\right)x\left(1-y\right)+\left(-L_{3}\right)\left(1-x\right)y+0\;\\ \bar{U_{3}}=zU_{3C}+\left(1-z\right)U_{3n}\; \end{array}\;$$

According to Equation (5), the replication dynamic equation of University researchers’ positive response to scientific research incentive is obtained:


(6) $$\begin{array}{l} F\left(z\right)=\frac{d_{z}}{d_{t}}=z\left(U_{3c}-\bar{U_{3}}\right)=\\ z\left(1-z\right)\left[\theta R_{3}x+\theta C_{2}y-C_{3}+L_{3}\left(x+y-xy\right)\right] \end{array}$$

Let $F\left(x\right)=\frac{d_{x}}{d_{t}}=0$ , $\;F\left(y\right)=\frac{d_{y}}{d_{t}}=0$ , $F\left(z\right)=\frac{d_{z}}{d_{t}}=0$ , and F(x)=F(y)=F(z) , combined with equations (2), (4), (6), eight equilibrium points E1 ~ E8 of the system conforming to the parameter value range are obtained, as shown in Table 3.

**Table 3:** Stable points of the tripartite evolutionary game among countries, universities, and researchers.

E1 (0, 0, 0)	E2 (0, 0, 1)	E3 (0, 1, 0)
E4 (0, 1, 1)	E5 (1, 0, 0)	E6 (1, 0, 1)
E7 (1, 1, 0)	E8 (1, 1, 1)	

Based on Equations (2), (4), and (6), the Jacobian matrix of the system is obtained as follows:


(7) $$J=\{\begin{array}{c} \left(1-2x\right)\left(L_{1}-C_{1}\right)\;0\;0\\ \begin{array}{c} \begin{array}{l} 0\;\left(1-2y\right)\left(L_{2}-\theta C_{2}z\right)\;\\ y\left(1-y\right)\left(-\theta C_{2}\right) \end{array}\\ \begin{array}{l} z\left(1-z\right)\left[\theta R_{3}+L_{3}\left(1-y\right)\right]\;\\ z\left(1-z\right)\left[\theta C_{2}+L_{3}\left(1-x\right)\right]\;\\ \left(1-2z\right)\left[\theta R_{3}x+\theta C_{2}y-C_{3}+L_{3}\left(x+y-\mathrm{xy}\right)\right] \end{array} \end{array} \end{array}$$

According to the equilibrium point stability judgment method proposed by Friedman (1991), the stability of the above eight equilibrium points is judged, and the results are shown in Table 4.

**Table 4:** Stability analysis of system equilibrium point.

Equilibrium point	det *J*	Symbol	tr *J*	Symbol	Stability
E1 (0, 0, 0)	$\left(-C_{3}\right)\left(L_{1}-C_{1}\right)L_{2}$	−	$L_{1}-C_{1}+L_{2}-C_{3}$	+	Unstable
E2 (0, 0, 1)	$C_{3}\left(L_{1}-C_{1}\right)\left(L_{2}-\theta C_{2}\right)$	+	$\left(L_{1}-C_{1}\right)+\left(L_{2}-\theta C_{2}\right)+C_{3}$	+	Unstable
E3 (0, 1, 0)	$\left(L_{1}-C_{1}\right)\left(-L_{2}\right)\left(\theta C_{2}+L_{3}-C_{3}\right)$	Uncertain	$\left(L_{1}-C_{1}\right)-L_{2}+\left(\theta C_{2}+L_{3}-C_{3}\right)$	+	Saddle point
E4 (0, 1, 1)	$\left(L_{1}-C_{1}\right)\left(L_{2}-\theta C_{2}\right)\left(\theta C_{2}+L_{3}-C_{3}\right)$	+	$L_{1}-C_{1}-\left(L_{2}-\theta C_{2}\right)-\left(\theta C_{2}+L_{3}-C_{3}\right)$	+	Unstable
E5 (1, 0, 0)	$-\left(L_{1}-C_{1}\right)L_{2}\left(\theta R_{3}-C_{3}+L_{3}\right)$	Uncertain	$-L_{1}+C_{1}+L_{2}+\theta R_{3}-C_{3}+L_{3}$	−	ESS (specific conditions)
E6 (1, 0, 1)	$\left(L_{1}-C_{1}\right)\left(L_{2}-\theta C_{2}\right)\left(\theta R_{3}-C_{3}+L_{3}\right)$	Uncertain	$-\left(L_{1}-C_{1}\right)+L_{2}-\theta C_{2}-\left(\theta R_{3}-C_{3}+L_{3}\right)$	−	ESS (specific conditions)
E7 (1, 1, 0)	$-\left(L_{1}-C_{1}\right)L_{2}\left(\theta R_{3}+\theta C_{2}-C_{3}+L_{3}\right)$	Uncertain	$-\left(L_{1}-C_{1}\right)-L_{2}+\left(\theta R_{3}+\theta C_{2}-C_{3}+L_{3}\right)$	−	ESS (specific conditions)
E8 (1, 1, 1)	$\left(L_{1}-C_{1}\right)\left(L_{2}-\theta C_{2}\right)\left(\theta R_{3}+\theta C_{2}-C_{3}+L_{3}\right)$	Uncertain	$-\left(L_{1}-C_{1}\right)-\left(L_{2}-\theta C_{2}\right)-\left(\theta R_{3}+\theta C_{2}-C_{3}+L_{3}\right)$	−	ESS (specific conditions)

According to the stability analysis of the above eight equilibrium points, there are four stable equilibrium points in the tripartite evolutionary game of the state, universities, and researchers, namely E5 (1,0,0), E6 (1,0,1), E7 (1,1,0), and E8 (1,1,1).

When $\theta <\frac{C_{3}-L_{3}}{R_{3}}$ , the three parties of the game have realistic dynamic stability at the E5 (1,0,0) equilibrium point. This stable point shows that the state actively encourages scientific research, but universities and researchers hold a negative attitude toward scientific research. Because scientific research innovation needs the active investment of scientific researchers, but the researchers at this stable point have a negative attitude toward scientific research, so this stable point is not the realistic goal we pursue.

When $\theta >\frac{C_{3}-L_{3}}{R_{3}}$ , the three parties of the game have realistic dynamic stability at the E6 (1,0,1) equilibrium point. This stable point shows that the state actively encourages scientific research, universities have a negative attitude toward scientific research incentives, and scientific researchers have a positive attitude toward scientific research. Although at this point, researchers will actively participate in scientific research, their work units and universities hold a negative attitude toward scientific research. Although the state has a positive policy on scientific research, the negative attitude of colleges and universities directly in charge of scientific research will weaken the scientific research enthusiasm of college teachers and affect the scientific research investment and scientific research innovation results of college teachers. Therefore, the stable point is not the best realistic goal we pursue.

When $\theta <\frac{C_{3}-L_{3}}{R_{3}+C_{2}}$ , the real dynamic stability of the three parties in the game at the E7 (1,1,0) equilibrium point. The practical significance of this stable point is that the state actively encourages scientific research and makes high investments in scientific research. Universities also actively encourage scientific research, but researchers treat scientific research negatively. In reality, only researchers actively participate in scientific research can create value for colleges and universities, and the state. Therefore, in reality, the stable point of E7 (1,1,0) is not our goal.

When $\theta >\frac{C_{3}-L_{3}}{R_{3}+C_{2}}$ , the three parties of the game have realistic dynamic stability at the equilibrium point of E8 (1,1,1). At this equilibrium point, the practical significance of the three parties of the game is that the state actively encourages scientific research and makes high investments in scientific research. Colleges and universities also actively encourage scientific researchers to participate in scientific research. In reality, only researchers actively participate in scientific research can create value for colleges and universities, and the state. Therefore, E8 (1,1,1) is our ideal goal in reality.

## 4. Numerical analysis and results

The above research shows that when $\theta <\frac{C_{3}-L_{3}}{R_{3}+C_{2}}$ , the three parties of the game have realistic dynamic stability at the equilibrium point E7 (1,1,0). At the equilibrium point E7 (1,1,0), the practical significance of the three parties of the game is that the state actively encourages scientific research and makes high investments in scientific research. Universities also actively encourage scientific research, but researchers treat scientific research negatively.

When $\theta >\frac{C_{3}-L_{3}}{R_{3}+C_{2}}$ , after a period, the final strategy of the three parties of the game is stable at E8 (1,1,1), that is, the dynamic stability point of the three parties of the game is that the state makes high investment in scientific research, university managers actively encourage scientific research, and scientific researchers actively participate in scientific research. To further explain the results of the tripartite evolutionary game, combined with the stable point conditions of the three parties of the game, the numerical simulation is carried out with MATLAB.

**Figure 1:**
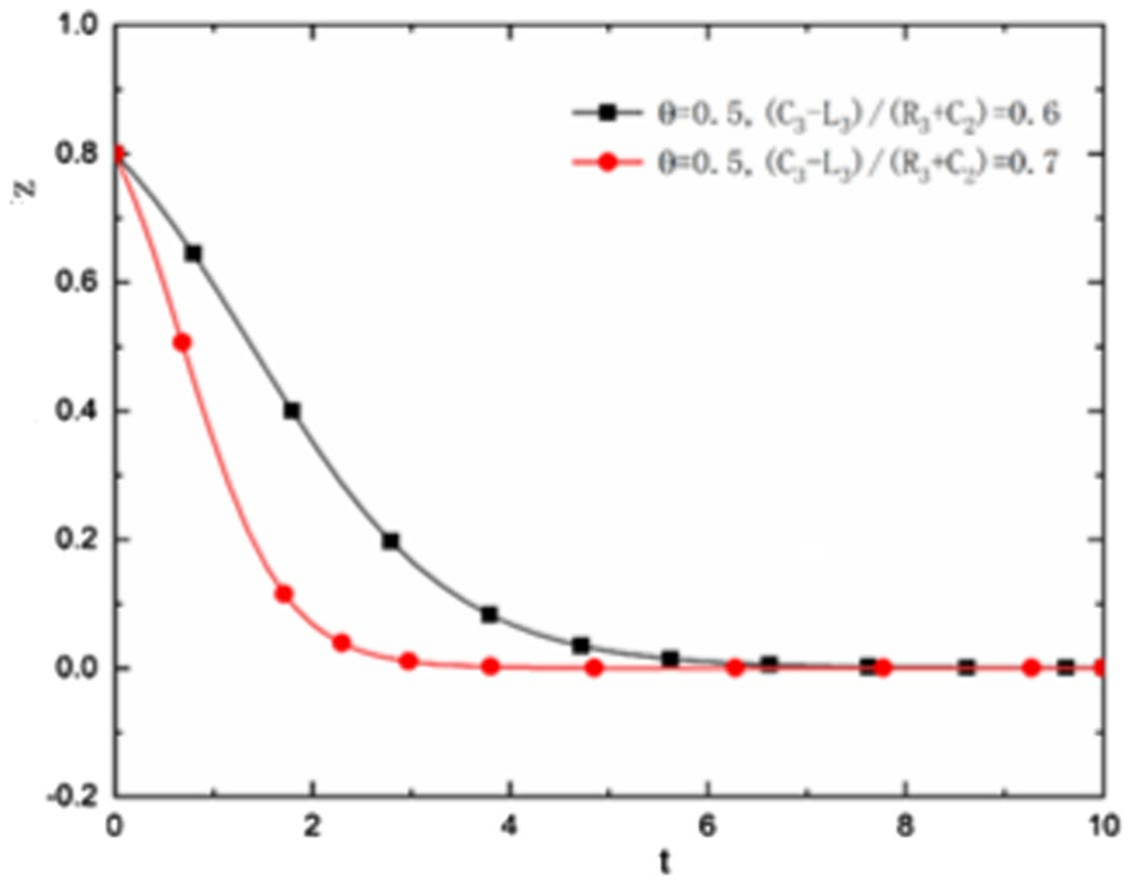
$\theta <\frac{C_{3}-L_{3}}{R_{3}+C_{2}}$ , the evolution trend of the three parties of the game.

1. According to the definition and assumptions of tripartite game variables (see Table 1 for details), the rate of scientific research output of University researchers is calculated θ is the cardinality. When θ=0.5 , it is assumed that the input–output ratio ( $\frac{C_{3}-L_{3}}{R_{3}+C_{2}})$ of University researchers are 0.6 and 0.7, respectively. Because the probability of scientific research success of University researchers is less than the rate of return on scientific research investment, $\theta <\frac{C_{3}-L_{3}}{R_{3}+C_{2}}$ . With time, due to more investment in scientific research, the state and universities have less incentive for scientific research, that is, The value of $C_{3}-L_{3}$ is very large, The value of $R_{3}+C_{2}$ is very small, resulting in a large value of $\frac{C_{3}-L_{3}}{R_{3}+C_{2}}$ , while the scientific research achievements of researchers are less, When the value θ is small, with time, the evolution result of the three parties of the game will gradually evolve to the trend that the state and universities do not actively encourage scientific research and researchers passively participate in scientific research. As shown in Figure 1.

In addition, Figure 1 also shows that in $\;\mathrm{the addition of}\;\theta <\frac{C_{3}-L_{3}}{R_{3}+C_{2}}$ , when there is little difference from the value of θ and $\frac{C_{3}-L_{3}}{R_{3}+C_{2}}$ , i.e., $\theta =0.5<\frac{C_{3}-L_{3}}{R_{3}+C_{2}}=0.6$ , the behavior evolution among the state, university managers, and University researchers needs a long time, and finally it is dynamically stable in the strategy that the state and universities do not encourage scientific research and researchers treat scientific research negatively. As shown by the black line in Figure 1. But if θ When it is very different from the value of $\frac{C_{3}-L_{3}}{R_{3}+C_{2}}$ , i.e., θ = When $\theta =0.5<\frac{C_{3}-L_{3}}{R_{3}+C_{2}}=0.7$ , the state, university managers, and University researchers will quickly change to the state and universities do not encourage scientific research, and researchers treat scientific research negatively. As shown by the red line in Figure 1.

**Figure 2:**
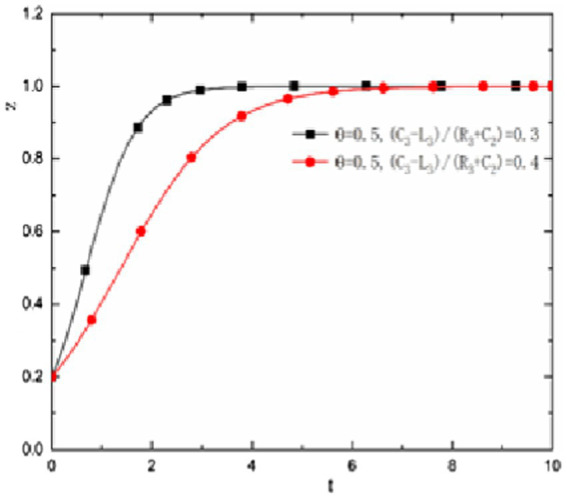
$\theta >\frac{C_{3}-L_{3}}{R_{3}+C_{2}}$ , the evolution trend of the three parties of the game.

2. When θ = 0.5, it is assumed that the input–output ratio $\frac{C_{3}-L_{3}}{R_{3}+C_{2}}$ of University researchers are 0.3 and 0.4, respectively. Because the probability of scientific research success of University researchers is greater than the return on investment in scientific research, that is $\theta >\frac{C_{3}-L_{3}}{R_{3}+C_{2}}$ . With time, due to less investment in scientific research, the state and universities have great incentives for scientific research, that is, the value of $C_{3}-L_{3}$ is very small, The value of $R_{3}+C_{2}$ is very large, resulting in the small value of $\frac{C_{3}-L_{3}}{R_{3}+C_{2}}$ , and the scientific research achievements of researchers are more, i.e., When the value θ is large, with time, the evolution results of the three parties of the game will gradually evolve to the trend that the state and universities actively encourage scientific research and scientific researchers participate in scientific research. As shown in Figure 2.

In addition, Figure 2 also shows that in $\theta >\frac{C_{3}-L_{3}}{R_{3}+C_{2}}$ , when θ When there is little difference from the value of θ and $\frac{C_{3}-L_{3}}{R_{3}+C_{2}}$ , i.e. $\mathrm{when}\;\theta =0.5>\frac{C_{3}-L_{3}}{R_{3}+C_{2}}=0.4$ , the behavior evolution among the state, university managers, and University researchers needs a long time, and finally the strategy of actively encouraging scientific research and actively treating scientific research by the state and universities is dynamically stable. As shown by the red line in Figure 2. But if it is very different from the value of θ and $\frac{C_{3}-L_{3}}{R_{3}+C_{2}}$ , i.e. When $\;\theta =0.5>\frac{C_{3}-L_{3}}{R_{3}+C_{2}}=0.3$ , the state, university managers, and University researchers will quickly actively encourage scientific research to the state and universities, and researchers will actively deal with the evolution of scientific research. As shown by the black line in Figure 2. The research conclusion shows that if we want scientific researchers to produce more, faster, and better results, in θ > In the case of $\;\theta >\frac{C_{3}-L_{3}}{R_{3}+C_{2}}$ , it needs to be improved the Ratio of θ and $\frac{C_{3}-L_{3}}{R_{3}+C_{2}}$ .

## 5. Conclusion and recommendations

According to the above three-party evolutionary game results, after a long-term game, both countries and universities will choose to actively encourage scientific research and innovation of scientific researchers, and whether scientific researchers will actively participate in scientific research and innovation depends on the relationship between the probability of scientific research and innovation achievements and the input–output ratio of investing a lot of time and energy to carry out corresponding scientific research and innovation. Only when the probability of achieving results is greater than the input–output ratio, University researchers will actively participate in scientific research, otherwise, they will give up scientific research and innovation because they invest too much but fail to achieve the expected results.

To effectively promote the scientific research enthusiasm of University researchers, first, we can improve the probability of scientific research innovation achievements by providing a better research environment and research conditions θ . Second, we can improve the national incentives for scientific research and innovation by raising the national awareness of respecting talents and knowledge $R_{3}$ . At the same time, improve the reward of colleges and universities for scientific research and innovation $C_{2}$ . Third, improve the research skills of researchers by enhancing their knowledge reserve, research literacy, and exchange learning, to reduce the research intensity $C_{3}$ . Fourth, we can also strengthen the assessment of University researchers who do not actively carry out scientific research work $\;L_{3}$ . However, excessively increasing the assessment strength will increase the hostility of scientific researchers, and its effect will be counterproductive. The adjustment of assessment strength should be fully considered, comprehensively measured, and carefully implemented. To sum up, to improve the scientific research enthusiasm of University researchers, priority should be given to the first three aspects mentioned above.

## Data availability statement

The original contributions presented in the study are included in the article/supplementary material, further inquiries can be directed to the corresponding author.

## Author contributions

The author confirms being the sole contributor of this work and has approved it for publication.

## Funding

This research was funded by the 14th five year plan project of Jiangxi Educational Science Planning, grant number (No. 22YB250); by the Science and Technology Project of Jiangxi Provincial Department of Education, grant number (No. GJJ211929); by the Humanities and Social Sciences Key Research Base Project of Universities in Jiangxi Province, grant number (No. JD20112); by the 2022–2023 topic of the Professional Committee of Talent Development of the China Education Development Strategy Society “Research on the Mechanism of the Intention of High level Talents Flow in Jiangxi Universities” [No. RCZWH2022021].

## Conflict of interest

The author declares that the research was conducted in the absence of any commercial or financial relationships that could be construed as a potential conflict of interest.

## Publisher’s note

All claims expressed in this article are solely those of the authors and do not necessarily represent those of their affiliated organizations, or those of the publisher, the editors and the reviewers. Any product that may be evaluated in this article, or claim that may be made by its manufacturer, is not guaranteed or endorsed by the publisher.
